# Short-chain fatty acids activate the glutamate-dependent acid resistance system via the tRNA-modifying Mnm pathway in *Escherichia coli*

**DOI:** 10.1128/jb.00285-26

**Published:** 2026-07-20

**Authors:** Lingyun Guo, Sophie Brameyer, Nicola Victoria Hoesch, Serena Schwenkert, Sebastián Riquelme-Barrios, Sabine Peschek, Max Berg, Manh Dat Hoang, Karin Kleigrewe, Stefanie Kaiser, Anna-Lena Heins, Kirsten Jung

**Affiliations:** 1 Faculty of Biology, Microbiology, Ludwig-Maximilians-University of Munich 543627, Martinsried, Germany; 2 Faculty of Biology, Mass Spectrometry of Biomolecules Service Unit, Ludwig-Maximilians-Universität München, Martinsried, Germany; 3 Goethe University Frankfurt, Faculty 14, Institute of Pharmaceutical Chemistry 9173, Frankfurt, Germany; 4 TUM School of Engineering and Design, Chair of Biochemical Engineering, Technical University of Munich, Garching, Germany; 5 Bavarian Center for Biomolecular Mass Spectrometry (BayBioMS), TUM School of Life Sciences, Technical University of Munich 84503 , Freising, Germany; Dartmouth College Geisel School of Medicine, Hanover, New Hampshire, USA

**Keywords:** acid resistance, Mnm pathway, glutamate decarboxylase, tRNA modification

## Abstract

Acid resistance is an important trait of enterobacteria, enabling survival under extreme gastric acidity (pH < 2.5) and the moderately acidic conditions in the large intestine (pH 5–6). In *Escherichia coli,* the glutamate-dependent acid resistance (Gad) system—comprising the glutamate decarboxylases GadA and GadB, and the antiporter GadC—is the major defense system against acid stress. Regulation of the Gad system is highly complex and involves multiple regulatory factors. In this study, we investigated the dynamics of *gadA*/*gadB* expression and GadA/GadB protein synthesis in *E. coli* MG1655 using a controlled bioreactor cultivation. Our results reveal that acid stress, oxygen limitation, and the stationary phase are the stimuli for Gad activation. Notably, acetic acid and other short-chain fatty acids (SCFAs) are superior stimuli of the Gad system than hydrochloric acid. Furthermore, we demonstrate that the tRNA-modifying MnmG/MnmE pathway responds to these SCFAs and stimulates Gad activation, likely via the transcriptional regulator GadW. These findings highlight a previously unrecognized link between SCFAs, tRNA modification, and acid stress resistance.

**IMPORTANCE:**

Survival of enteric bacteria in the gastrointestinal tract depends on their ability to sense and respond to diverse forms of acid stress. This study identifies oxygen limitation, growth phase, and—most importantly—short-chain fatty acids (SCFAs) as key physiological triggers of the major acid resistance system in *Escherichia coli*. In addition, we uncover a previously unrecognized connection between SCFAs-sensing, tRNA modification via the MnmG/MnmE pathway, and activation of acid resistance, providing new insight into how metabolic and environmental signals are integrated to promote bacterial survival.

## INTRODUCTION

To successfully colonize the gastrointestinal tract of vertebrates, enterobacteria such as *Escherichia coli* must withstand both extreme acid stress in the stomach (pH < 2.5), primarily caused by hydrochloric acid, and moderate acid stress in the colon (pH 5.0–6.5), caused by short-chain fatty acids (SCFAs). SCFAs, such as acetate, propionate, and butyrate, are produced through fermentation by obligate anaerobic members of the gut microbiota (1–4). To survive in these conditions, *E. coli* has several sophisticated acid resistance (AR) systems, including three proton-consuming amino acid decarboxylase systems: the glutamate decarboxylase (Gad) system, the arginine decarboxylase (Adi) system, and the lysine decarboxylase (Cad) system (also referred to as AR 2, AR 3, and AR 4, respectively). The function and regulatory complexity of these systems have been extensively reviewed (5–10).

We previously demonstrated that the *E. coli* population exhibits functional diversification to withstand consecutively increasing acid stress (11). Using a fluorescent triple reporter strain, we observed that all cells more or less activate the Gad system, and distinct subpopulations additionally induce either the Cad or Adi systems (11). Notably, the Gad system is essential for the survival of *E. coli* at an extremely low pH of 2.5 (12–15) and is therefore induced in all cells of the population. This study was undertaken to elucidate the primary stimuli that govern activation of the Gad system.

The Gad system consists of two homologous inducible glutamate decarboxylases, GadA and GadB, and the glutamate/γ-aminobutyric acid (GABA) antiporter GadC (12–15). The *gadB* and *gadC* genes are co-transcribed, while the *gadA* gene, located 2.1 Mb away from *gadBC*, is transcribed either alone or co-transcribed with one of the regulatory genes, *gadX* (16, 17). *gadA* and most Gad regulatory genes, such as *gadX*, *gadY*, *gadW,* and *gadE,* are located in the acid fitness island (AFI) (18, 19). The induction of *gadA* and *gadBC* depends on environmental pH, growth phase, and oxygen availability (14, 16, 20).

The regulatory network of the Gad system is extremely complex and includes transcription factors, two-component signal transduction systems (TCSs), H-NS, and small RNAs. The LuxR-family transcription factor GadE is the central regulator of the Gad system. It can bind directly to the 20 bp GAD box, which is located in the promoter regions of *gadA* and *gadBC* (21). RcsB, the response regulator of the Rcs (Regulator of Capsule Synthesis) TCS, is essential for the expression of *gadA* and *gadB* and forms functional heterodimers with GadE (22–24).

There are three major regulatory circuits/pathways—EvgS/EvgA, GadX/GadW, and Mnm—that regulate the expression of the central transcription regulator GadE, which, in turn, activates *gadA* and *gadBC* expression (5, 25). (i) The EvgS/EvgA circuit consists of the histidine kinase EvgS, the response regulator EvgA, and the AraC-like regulator YdeO (26, 27). EvgS is activated by moderately acidic pH in the presence of alkali metals (Na^+^ or K^+^) during the exponential growth phase. The signals are then transduced either from EvgS to EvgA to YdeO to GadE, or directly from EvgA to GadE without YdeO (28, 29). Recent studies have identified an extension of the EvgS/EvgA circuit by the PhoQ/PhoP TCS that responds to external Mg^2+^. The activated EvgS/EvgA system activates the PhoQ/PhoP system via the connector protein SafA (Sensor-associating-factor A). Once active, the PhoQ/PhoP system induces another connector, IraM, which binds directly to the recognition factor RssB and prevents proteolysis of the stationary σ-factor RpoS. RpoS activates *gadE* expression (30–33). (ii) The second circuit, GadX/GadW, comprises the two AraC-like transcription regulators GadX and GadW (34), the stationary σ-factor RpoS (14), the cyclic AMP receptor protein CRP (34), and the small RNA GadY (35). The GadX/GadW circuit conditionally activates *gadA* and *gadBC* expression indirectly by activating *gadE* (25) or represses *gadA* and *gadBC* by directly binding to the GAD box in the promoter regions of *gadA* and *gadBC* (17, 34). It has been proposed that GadX, GadW, and CRP sense different chemical stimuli and that the intracellular ratio of these signals alters the balance in this regulatory circuit (5). (iii) The third pathway is the Mnm pathway. This evolutionarily conserved pathway modifies the wobble uridine (U_34_) in specific tRNAs (tRNA^Lys^, tRNA^Glu^, tRNA^Gln^, tRNA^Arg^, tRNA^Leu^, and tRNA^Gly^) to improve translation efficiency and decoding accuracy of NNA/G codons. The most common final modifications are 5-methylaminomethyl-2-thiouridine (mnm^5^s^2^U_34_) and 5-methylaminomethyluridine (mnm^5^U_34_). MnmA catalyzes the thiolation of U34 (s^2^U_34_ at the 2′ position). The MnmE-MnmG complex (also known as TrmE-GidA) catalyzes the addition of an aminomethyl (nm) or carboxymethylaminomethyl (cmnm) group to the 5′ position of U_34_. The bifunctional enzyme MnmC converts the MnmEG products into the mnm^5^s^2^U and nm^5^s^2^U modifications (36–40). It has previously been reported that MnmE (TrmE) and GadE are both essential for GadA/B production in *E. coli* grown in tryptone/glucose medium (pH 5.2) (41).

In this study, we used a controlled bioreactor cultivation on a laboratory scale and showed that Gad activation is driven by acid stress, oxygen limitation, and the stationary phase. We found that excreted extracellular acetate and other SCFAs are stronger inducers than hydrochloric acid (HCl). Furthermore, SCFAs sensing was found to be linked to the Mnm-dependent tRNA modification and affects Gad regulation, likely via GadW.

## RESULTS

### The time-resolved activation profile of the Gad system in *E. coli* MG1655 grown in a stirred-tank bioreactor at pH 7.6

**Fig 1:**
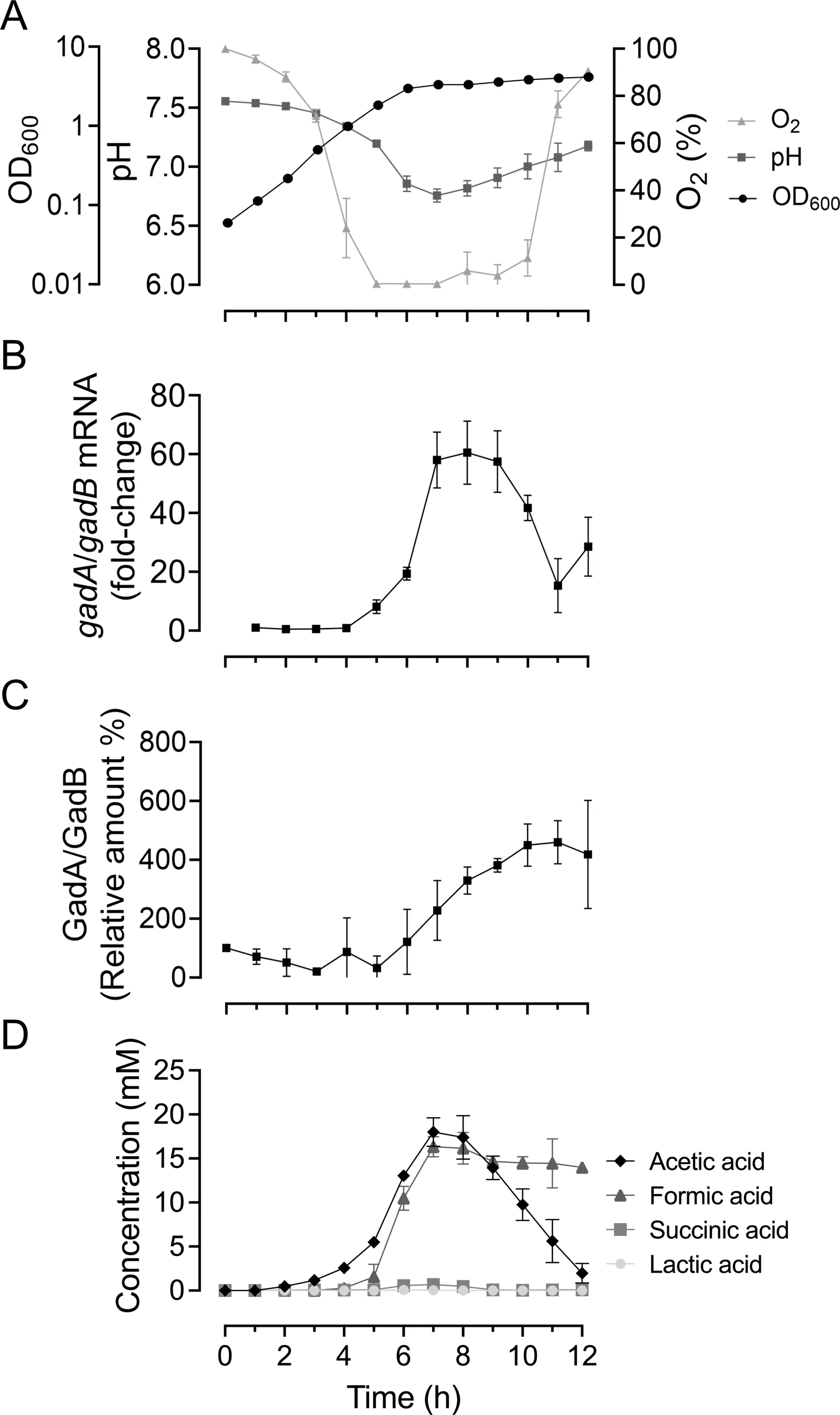
Time-resolved expression profile of the Gad system in *E. coli* MG1655 during cultivation in a well-stirred bioreactor. Exponentially grown *E. coli* was used to inoculate a 900 mL bioreactor at time 0 and was further cultivated in KE glucose-glutamate minimal medium at pH 7.6 and 37°C. At the indicated times, samples were taken and analyzed. (**A**) Growth of *E. coli* (OD_600_, black) over time. The medium pH (gray) and dissolved oxygen (light gray) were measured using bioreactor electrodes. (**B**) Time-resolved dynamics of *gadA/gadB* mRNA analyzed by qRT-PCR. The 16S rRNA gene was used as the internal reference, and fold-change was normalized to time 1 h. To keep the Ct value within the same range, the template cDNA used to measure the 16S rRNA gene was diluted 10,000-fold compared to the sample used to measure *gadA/gadB* mRNA. (**C**) Time-resolved dynamics of GadA/GadB measured by Western Blot. The amount of GadA/GadB produced was analyzed by Western Blot using a specific anti-GadA/GadB antibody. A total of 16 μL of cells adjusted to an OD_600_ of 10 was loaded per lane. The SDS gel was stained with 2,2,2-trichloroethanol (TCE) and used as a loading control. Blots were scanned at high resolution in 256 gray scales and analyzed by ImageQuant 5.0. Values were normalized to time 0. (**D**) Time-resolved dynamics of external metabolites. Quantification of external metabolites was performed using high-performance liquid chromatography (HPLC). Experiments were performed in biological triplicate. Error bars represent the standard deviation of the means.

To elucidate the nature of the primary stimuli that activate the Gad system, we adopted a strategy that we had previously used for the Cad system (42). Instead of fluorescent reporter strains and endpoint determinations of *E. coli* cells grown under different conditions, we monitored time-resolved changes in wild-type *E. coli* under the defined conditions of a bioreactor. Therefore, we cultivated *E. coli* MG1655 in buffered minimal medium (pH 7.6) with glucose (0.4%) as a carbon source and glutamate (10 mM) in a bioreactor with 200 rpm air agitation at 37°C. Samples were taken every 60 min to quantify the levels of *gadA/gadB* mRNA and GadA/GadB proteins, as well as optical density (OD_600_), pH, dissolved oxygen level, and four major external metabolites (Fig. 1). What we initially considered as a control experiment became an ideal setup to study the induction of the Gad system, because unexpectedly, in the middle of the exponential growth phase, the dissolved oxygen level declined, probably due to the high demand of an increasing cell number, and was depleted after 5 h of growth (Fig. 1A). Shortly afterward, the pH dropped to 6.8, and the growth slowed down (Fig. 1A). With the lower growth rate, oxygen consumption by the culture decreased, and, due to constant aeration by stirring, the dissolved oxygen level increased. A similar pattern of drop in dissolved oxygen level and decrease of pH has been previously described for the cultivation of *E. coli* in glucose minimal medium (43, 44). The drop in dissolved oxygen level, accompanied by the decrease in pH to 6.8 and the transition to the stationary phase, was sufficient to activate the expression of *gadA/gadB* mRNA (after 5 h); the transcript level increased and remained high until 9 h of growth (Fig. 1B). Immediately, GadA/GadB proteins could be detected in the Western blot (Fig. 1C). Furthermore, due to the limitation in oxygen, *E. coli* had switched to mixed acid fermentation, which correlated with the increase in the major external metabolites, acetic acid, formic acid, and a small amount of succinic acid, while lactic acid was not detectable (Fig. 1D). Excretion of acetic acid started during the exponential growth phase of *E. coli* and reached a maximum value of 20 mM in the stationary phase (time point 7 h). After that, it was reassimilated by *E. coli* (Fig. 1D). Besides fermentation, the dynamics of acetic acid can also be explained by the acetate switch (45), as *E. coli* metabolizes glucose incompletely, dissimilates acetate, and takes it up later. The reuptake of acetic acid was accompanied by an increase in pH to 7.2 in the stationary phase (Fig. 1A). *E. coli* also secreted formic acid as a fermentation product; its concentration reached the maximum of 15 mM in the stationary phase (time point 7 h) and remained at this level until the end of the experiment (Fig. 1D). It should be noted that the cultivation was performed in phosphate-buffered medium (pH 7.6), and theoretical calculations using the Henderson-Hasselbalch equation revealed that the addition of 20 mM acetic acid and 20 mM formic acid lowers the pH by 0.8 units, which matches our experimental measurement (pH 6.8).

In summary, these results reveal that the Gad system is even activated in *E. coli* when grown in a buffered medium (pH 7.6) in a stirred-tank bioreactor due to both a transient oxygen limitation and a subsequent lower extracellular pH (about pH 6.8). In addition, organic acids were excreted, and the culture transitioned into the stationary phase. These results underline once more that the Gad system is activated in response to anaerobiosis, low pH, and the stationary phase, which is in line with previous reports from other labs (14, 16, 20).

### Acetic acid is the dominant extracellular metabolite under Gad-inducing conditions

**Fig 2:**
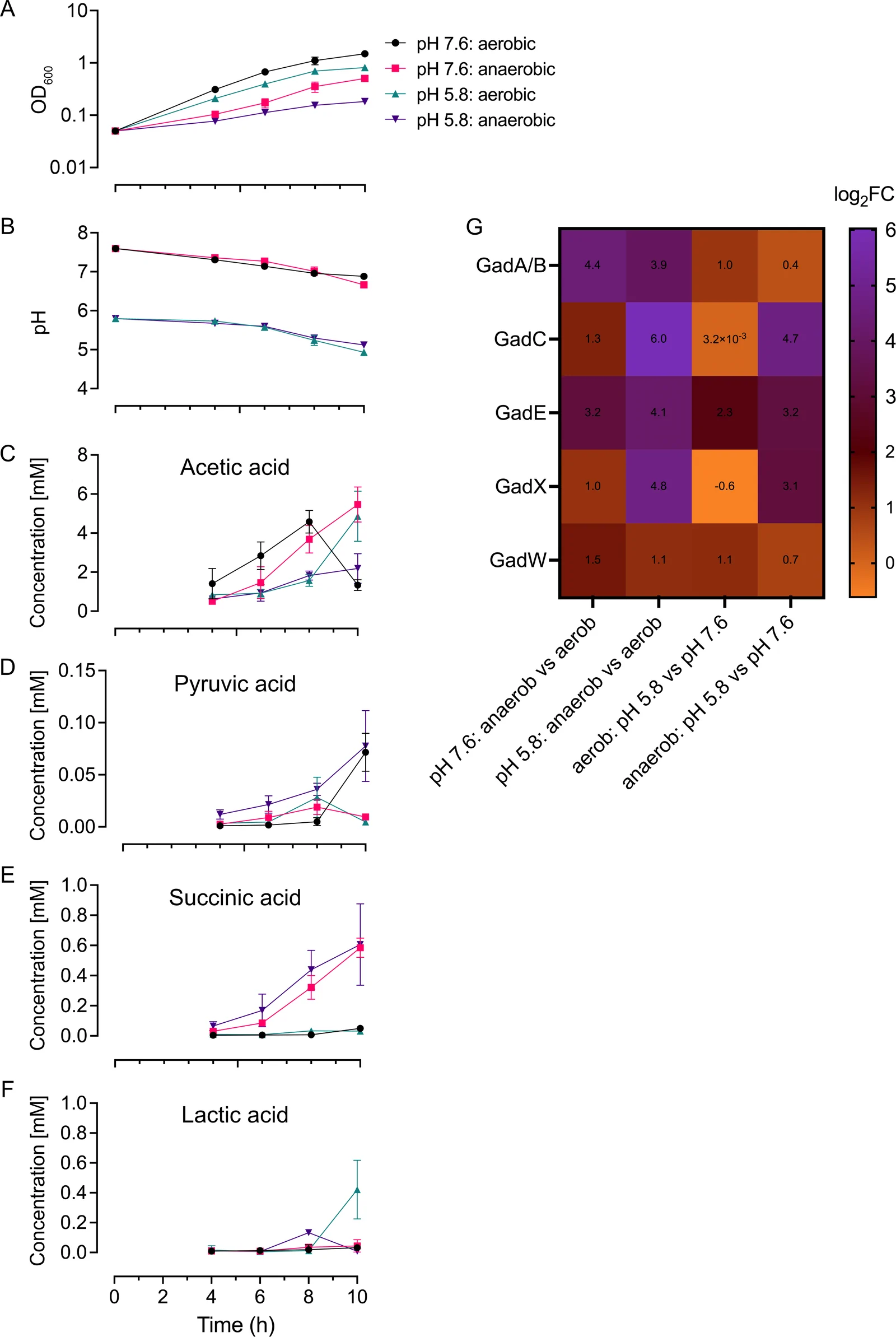
Effect of oxygen availability and acidity on the growth, extracellular pH, excreted metabolites, and components of the Gad system in *E. coli* MG1655. *E. coli* MG1655 was grown in KE medium (phosphate buffered at pH 7.6 or 5.8) supplemented with 0.4% (wt/vol) glucose and 1.5 mM glutamic acid under aerobic (baffled flask, 200 rpm) and anaerobic (Falcon tube, standing) conditions. Growth (OD_600_) (**A**), extracellular pH (**B**), and excretion of acetic acid (**C**), pyruvic acid (**D**), succinic acid (**E**), and lactic acid (**F**) were followed over time. After 6 h of growth, the proteome was analyzed (**G**). Shown is the heatmap of log_2_ fold changes (log_2_FC) from the proteomics data for the major functional and regulatory components of the Gad system. Protein levels of anaerobic versus aerobic conditions were compared at either pH 7.6 or pH 5.8. Numbers in tiles denote the log_2_FC values, and the color scale visualizes the magnitude of change. Data represent the mean and standard deviation for three individual biological replicates. Statistical significance was assessed using a two-tailed, unpaired t-test with permutation-based FDR correction applied (S0 = 0.1, *P* ≤ 0.05, Data set S1). Because the two glutamate decarboxylase isoenzymes GadA and GadB of *E. coli* MG1655 share 98.9% amino acid sequence identity, they were grouped in the proteome analysis.

We next examined in more detail the influence of the extracellular pH and oxygen availability on the excretion of metabolites and the components of the Gad system in *E. coli* MG1655. To simplify the approach, we cultivated *E. coli* in shake flasks under aerobic conditions or in closed top-filled Falcon tubes under anaerobic conditions in phosphate-buffered medium at pH 7.6 or pH 5.8 as described above, and compared growth, extracellular pH, metabolite formation, and the major components of the Gad system (Fig. 2). As expected, cells grew best at pH 7.6 under aerobic conditions; the lowest OD_600_ was observed under anaerobic acidic conditions (Fig. 2A). In all cultures, an acidification was observed over time: the pH of the pH 5.8-culture declined to approximately 5.0–5.1 after 10 h of growth, and the pH of the pH 7.6-culture declined to approximately 6.7–6.9 (Fig. 2B). Strikingly, *E. coli* excreted high amounts of acetic acid under all conditions, which reached extracellular concentrations between 1 and 6 mM (Fig. 2C). In contrast, pyruvic acid was hardly excreted (below 0.1 mM) (Fig. 2D). Succinic acid and lactic acid (Fig. 2E and F), two fermentation products, were excreted by *E. coli* grown under anaerobic conditions at low concentrations (succinic acid, a maximum of 0.6 mM, lactic acid 0.1 mM). In the late growth stage of the aerobic pH 5.8-culture, for unknown reasons, slightly more lactic acid (0.4 mM) was detectable.

After 6 h of growth, we collected samples from all four cultures to profile the abundance of the functional and regulatory components of the Gad system using a proteomic approach (Fig. 2G). The decarboxylases GadA and GadB were strongly upregulated under all conditions, with their levels increased in the anaerobic cultures at both pH 7.6 and pH 5.8. The level of the central transcriptional activator GadE mirrored the pattern of GadA/GadB, while the antiporter GadC and the transcriptional regulator GadX showed particularly pronounced anaerobic synthesis at pH 5.8 (Fig. 2G). The GadW level changed less, but was highest under anaerobic conditions at a pH of 7.6 (Fig. 2G).

Together, these data show that the Gad acid resistance network is activated under all conditions, consistent with our bioreactor experiment (Fig. 1). However, the levels of individual components of the Gad system are fine-tuned by external parameters such as pH and/or oxygen availability, which also influence the catabolic activity of *E. coli*.

The complete proteome data sets are available, but are not described in detail here for clarity (Data Set S1).

### SCFAs demonstrate superior activation of the Gad system over hydrochloric acid

We found that excretion of acetic acid by *E. coli* coincides with the activation of the Gad system (Fig. 1 and 2). In addition to self-produced organic acids, *E. coli* encounters acid stress caused by SCFAs, such as propionic acid and butyric acid, which are produced during fermentation by other obligate bacteria in the human colon (1, 2). Pretreatment of *E. coli* with SCFAs has been shown to increase acid tolerance at pH 3.0 (46). Based on these results, we hypothesized that SCFAs serve as external stimuli for the Gad system in *E. coli*.

The transcripts of *gadB* and *gadA* are almost indistinguishable, with about 97.8% sequence identity. *gadB*, located at 33 min (1.57 Mb), is a product of gene duplication, while *gadA* is located at 78 min (3.67 Mb) on the chromosome as part of the acid fitness island. Their upstream regulatory sequences are similar but not identical (47). To test the influence of SCFAs on *gadB* and *gadA* induction, we constructed two promoter-fluorophore fusions, P*_gadB_:mcherry* and P*_gadA_:egfp*. Both fusions were inserted into a single plasmid, pBBR1-MCS4-P*_gadB_:mcherry*-P*_gadA_:egfp*, to distinguish activation of the promoters of either *gadB* or *gadA* using the two fluorophores.

**Fig 3:**
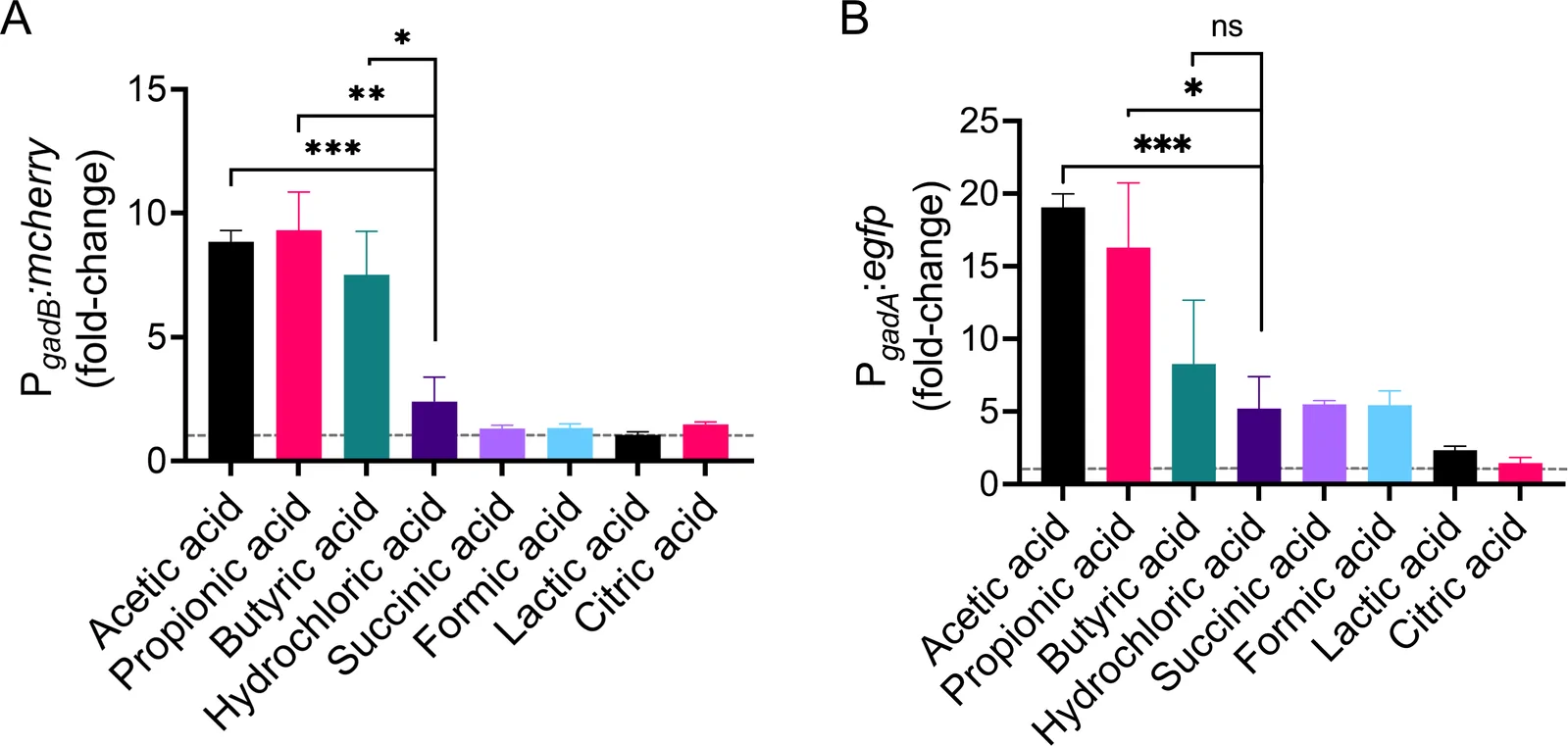
The effect of different acids on *gadB* and *gadA* promoter activity in *E. coli* MG1655. (**A**) The effect of different acids on *gadB* promoter activity. (**B**) The effect of different acids on the *gadA* promoter activity. *E. coli* MG1655 cells were transformed with a plasmid-based fluorescent promoter fusion of P*_gadB_:mcherry*-P*_gadA_:egfp*. 50 mM acid was added to the cultures after 5 h of incubation (exponential growth phase) with the starting OD_600_ of 0.05 in glycerol glutamate minimal medium (pH 7.6). *gadB* and *gadA* promoter activities were tested by measuring fluorescence and OD_600_ using a plate reader after 3 h of incubation. Fold change values were normalized to the non-inducing condition (no acid added, dashed gray line). Data represent the mean and standard deviation of three independent biological replicates. Statistical analysis was performed using a two-tailed unpaired t-test. *P ≤ 0.05, **P = 0.00218, ***P ≤ 0.0005, ns, non-significant, *P* > 0.05.

To prevent any effects from the stationary phase and oxygen limitation, we exposed exponentially growing *E. coli* MG1655 transformed with the reporter plasmid (after 5 h of cultivation in pH 7.6 buffered mineral medium with glycerol as the carbon source and glutamate) to the SCFAs acetic, propionic, and butyric acids, and as well as other organic acids, namely succinic, lactic, formic, and citric acids (each at 50 mM). We used glycerol as the carbon source instead of glucose to avoid acetate production. The activity of the *gadB* promoter (Fig. 3) significantly increased after exposure to SCFAs, such as acetic, butyric, and propionic acid, compared to hydrochloric acid (HCl). Succinic acid, formic acid, lactic acid, and citric acid only marginally induced the *gadB* promoter. The *gadA* promoter was significantly induced by acetic and propionic acid, but less so by butyric acid (Fig. 3B). Exposure to succinic acid and formic acid led to an increase in *gadA* promoter activity similar to that observed with HCl. It is important to note that the addition of 50 mM of each acid lowered the medium pH to similar values (acetic acid pH 6.64; propionic acid pH 6.61; butyric acid pH 6.71; succinic acid pH 5.97; lactic acid pH 6.61; formic acid pH 6.55; HCl pH 6.62). The only exception was citric acid, which lowered the pH to 4.57; nevertheless, it hardly activated the *gadB* or *gadA* promoter, and the value was lower than for HCl (Fig. 3).

These results reveal that the promoters of *gadB* and *gadA* are not only activated by a decrease in external pH, but specifically by SCFAs.

### SCFAs activate the Gad system via the Mnm pathway

**Fig 4:**
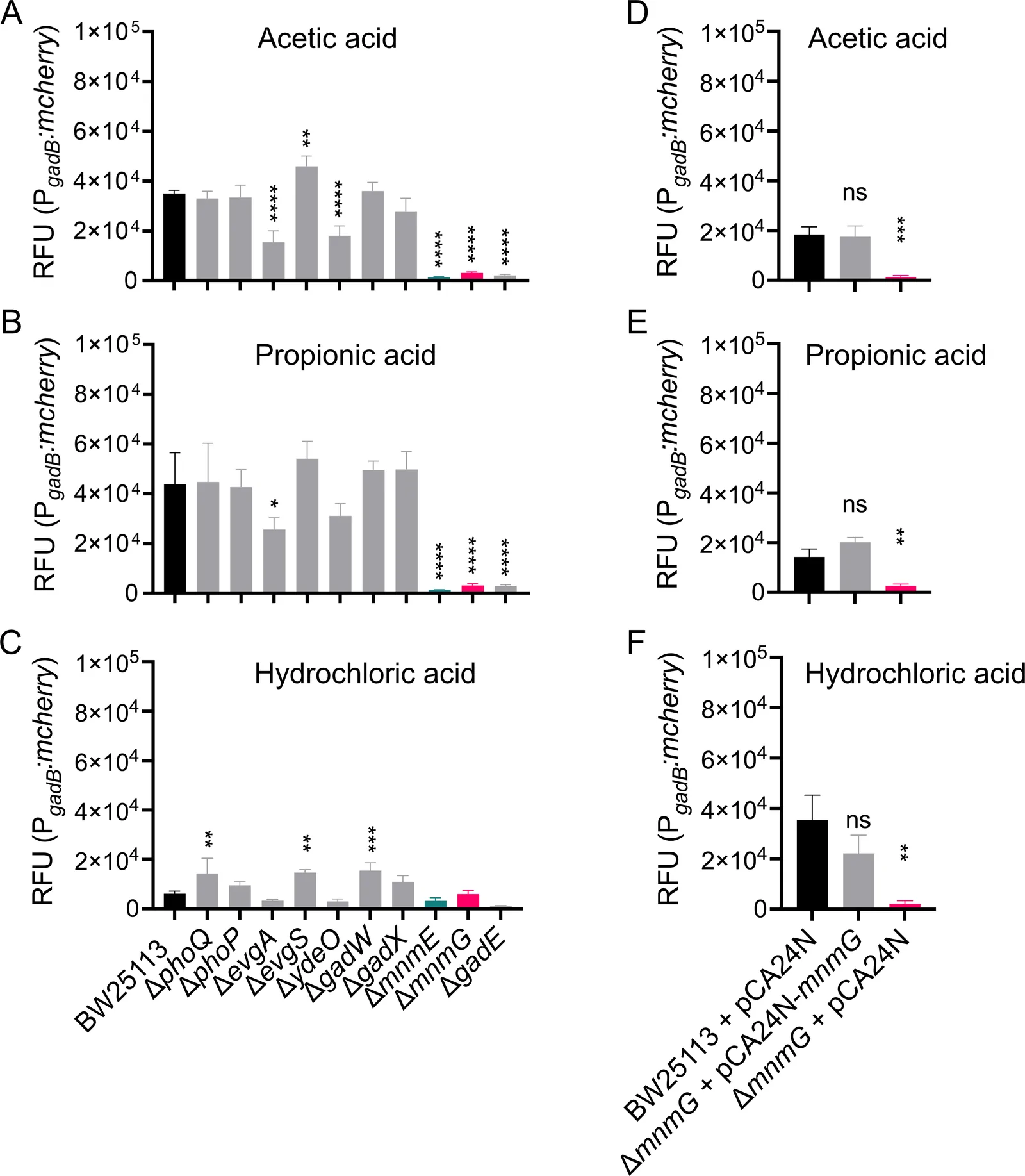
Screening of regulatory mutants of the Gad system for effects on *gadB* promoter activity after exposure to SCFAs. The *gadB* promoter activity was tested in *E. coli* BW25113 and various isogenic mutants after exposure to different acids: (**A**) acetic, (**B**) propionic, and (**C**) hydrochloric acid. The culture and test conditions were as described in Fig. 3. To assess complementation of the *E. coli* BW25113 *mnmG* mutant, *E. coli* BW25113, the *E. coli* BW25113 *mnmG* mutant carrying the empty pCA24N vector, and the *E. coli* BW25113 *mnmG* mutant carrying pCA24N-*mnmG* were cultivated as described in Fig. 3, except that glucose replaced glycerol. After 3 h of incubation, when external acetate levels were still low, cells were exposed to (**D**) acetic acid, (**E**) propionic acid, or (**F**) hydrochloric acid. Promoter activity was determined as RFU, defined as relative fluorescence units divided by OD_600_. Experiments were performed in biological replicates. Error bars represent the standard deviation of the means. Statistical analysis was performed using Dunnett’s multiple comparison test, comparing all strains to the *E. coli* BW25113 data, *P ≤ 0.05, **P ≤ 0.01, ***P ≤ 0.0009, ****P ≤ 0.0001, ns, non-significant, *P* > 0.05.

In our next experiments, we aimed to identify the regulatory pathway responsible for activation of the Gad system by SCFAs. Therefore, the P*_gadB_:mcherry* reporter was analyzed in *phoQ*, *phoP*, *evgA*, *evgS*, *ydeO*, *gadW*, *gadX*, *mnmE*, and *mnmG E. coli* BW25113 mutants exposed to acetic acid or propionic acid during the exponential growth phase. Exposure to HCl served as a control condition. For this screen, the *gadE* mutant and the parental strain *E. coli* BW25113 were used as the negative and positive control strains, respectively. Of all mutants tested, the *mnmG* and *mnmE* mutants showed the lowest *gadB* promoter activity in response to acetic acid and propionic acid, which was in the range of the *gadE* mutant (Fig. 4A through C). Additionally, the *evgA* and *ydeO* mutants exhibited reduced *gadB* promoter activity compared with the wild type in response to acetic acid and propionic acid (Fig. 4A and B), although this reduction was less pronounced than in the *mnmE* and *mnmG* mutants.

The *mnmG* mutant was complemented *in trans* using plasmid pCA24N-*mnmG* under the control of the P_T5-*lac*_ promoter and co-transformed with the reporter plasmid pBBR1-MCS4-P*_gadB_:mcherry*-P*_gadA_:egfp* to analyze *gadB* promoter activation in response to different acids. Because the two-plasmid system caused a major growth defect in *E. coli* BW25113 in the glycerol-glutamate minimal medium, glucose was used as the carbon source instead of glycerol. To minimize the effect of mixed acid fermentation products of glucose, we exposed *E. coli* to acid stress after 3 h of incubation, when the external acetate concentration was still low (Fig. 1D). Activation of *gadB* promoter in response to the SCFAs, propionic and acetic acid, and HCl, was fully restored in the complemented *mnmG* mutant (Fig. 4D through F), confirming that the Mnm pathway plays a significant role in the regulation of the *gadB* promoter in response to the SCFAs.

### The Mnm pathway influences the copy number of GadA/B and GadW

**Fig 5:**
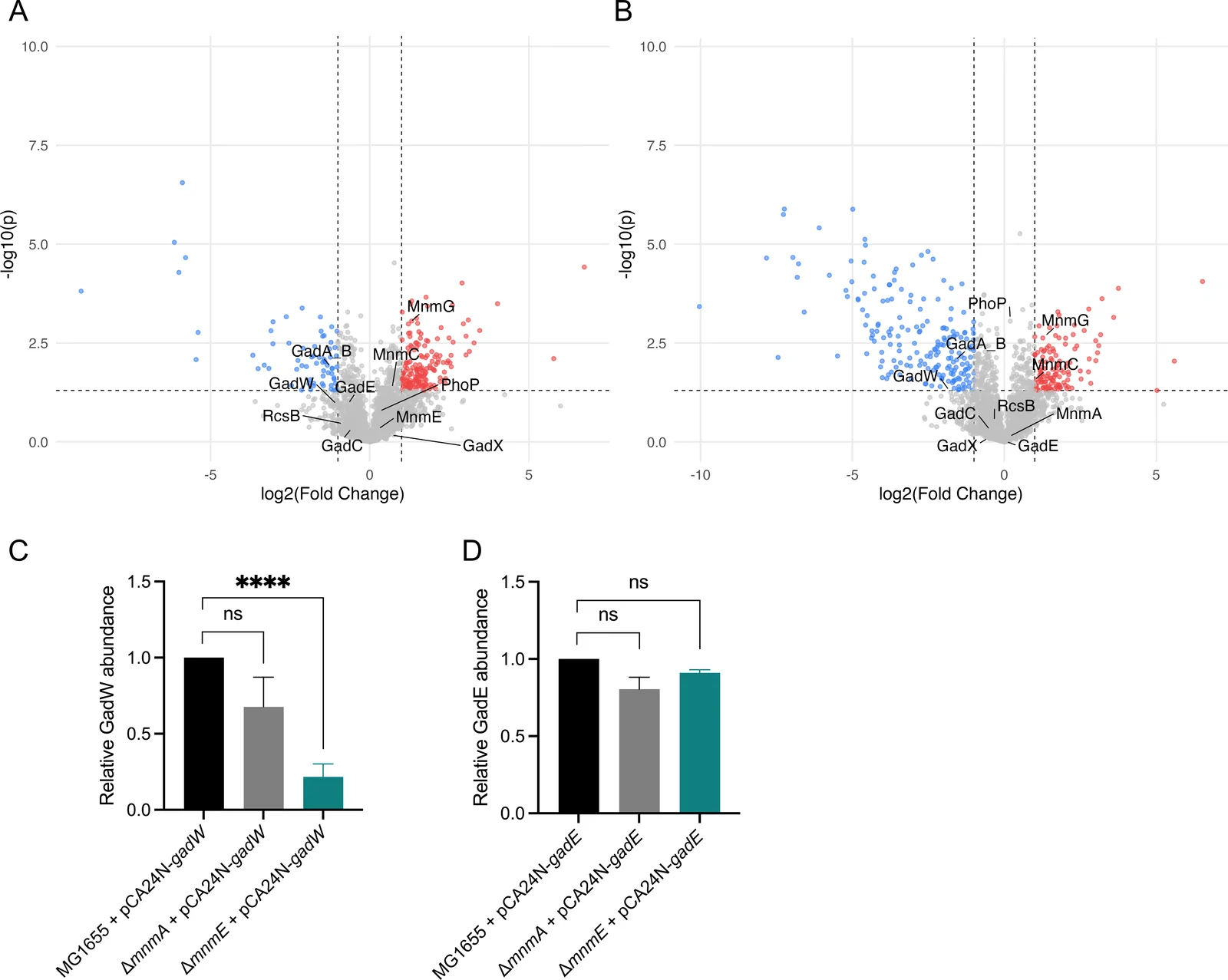
Differential proteome analysis of *E. coli* MG1655 *mnmA* and *mnmE* mutants relative to the parental strain. Volcano plots show changes in protein abundance in the *mnmA* mutant (**A**) and the *mnmE* mutant (**B**) compared with wild-type *E. coli* MG1655. Cells were cultivated in KE medium at pH 5.8, supplemented with glucose and glutamate, under anaerobic conditions, and harvested after 6 h of incubation. The x-axis shows log_2_ fold change, and the y-axis shows −log10(*P*). A two-tailed unpaired student’s *t*-test with permutation-based FDR correction (FDR = 0.05, S0 = 0.1) was performed. Dashed vertical lines mark the fold-change cutoff at log_2_FC = ±1, corresponding to a twofold change, and the dashed horizontal line marks the significance cutoff at −log10(*P*) = 1.301 (*P* = 0.05). Downregulated proteins are shown in blue, upregulated proteins in red, unchanged proteins in gray. Statistically significant changes are indicated in Data set S1. Selected proteins of interest, including components and regulators of the Gad acid-resistance system, are highlighted. Both mutants exhibited reduced abundance of Gad-associated proteins, with stronger effects in the *mnmE* mutant. Loss of MnmA and MnmE was confirmed by the decreased abundance of the corresponding proteins in the respective mutant backgrounds. Data represent three biological replicates per strain (*n* = 3). Because the two glutamate decarboxylase isoenzymes GadA and GadB of *E. coli* MG1655 share 98.9% amino acid sequence identity, they were grouped in the proteome analysis. (**C and D**) Bars show the relative abundance of GadW (**C**) and GadE (**D**) measured in *E. coli* MG1655 wild type (black), *mnmA* mutant (gray), and *mnmE* mutant (green) via quantitative Western blot analysis. Cells were cultivated anaerobically in KE medium at pH 5.8 supplemented with glucose and glutamate for 3 h. IPTG was then added to a final concentration of 100 µM, and cells were harvested after an additional 1 h of incubation. Data represent three biological replicates per strain (*n* = 3), and statistical significance was determined using a two-tailed unpaired *t*-test. ****P ≤ 0.0001, ns, not signficant, *P* >0.05.

Given the strong contribution of the Mnm pathway to *gadB* promoter activation, we constructed *mnmA* and *mnmE* deletion mutants in the *E. coli* MG1655 background. Then, we compared the proteome of the *mnmA* and *mnmE* mutants relative to the *E. coli* MG1655 parental strain under anaerobic, acidic conditions (pH 5.8). In both mutants, the glutamate decarboxylases GadA and GadB were downregulated, with the effect more pronounced in the *mnmE* mutant than in the *mnmA* mutant (Fig. 5A and B). Notably, the transcriptional regulator GadW, and not GadE, was downregulated in the *mnmE* mutant compared with the wild type (Fig. 5B). This observation was validated by Western blot analysis using His-tagged GadW, which confirmed a significant decrease in GadW abundance in the *mnmE* mutant, while GadW levels in the *mnmA* mutant remained largely unchanged (Fig. 5C). In contrast, GadE abundance was unaffected in either mutant validated by Western blot analysis using His-tagged GadE (Fig. 5D). Interestingly, MnmG was upregulated in both mutants relative to the parental strain (Fig. 5A and B), suggesting a potential compensatory response within the Mnm circuit that contributes to the regulation of the Gad system. The complete proteome data sets are available (Data Set S1), but are not described in detail here for clarity.

### The mnm^5^s^2^U_34_-tRNA level increases under anaerobic conditions

**Fig 6:**
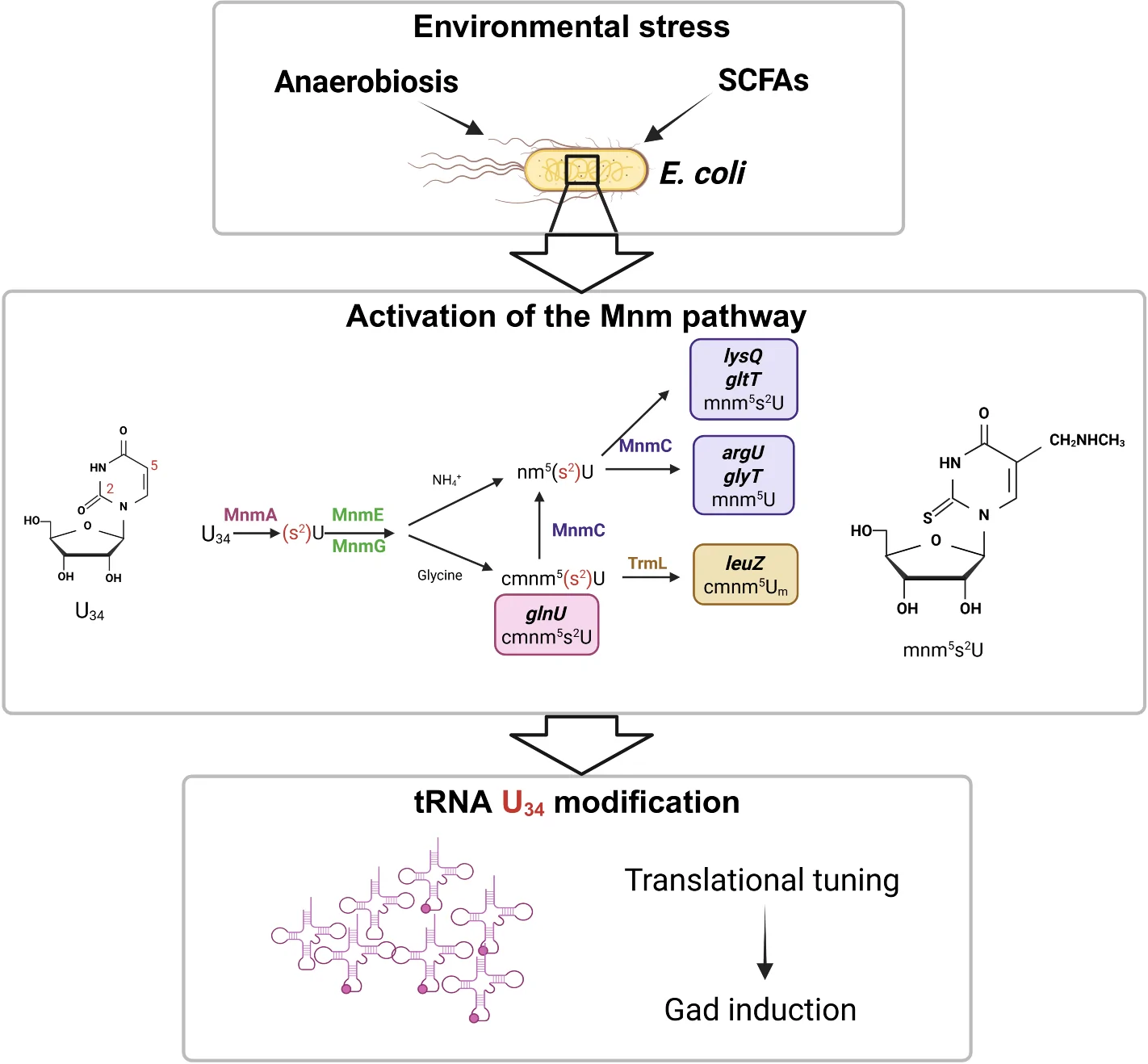
Model for activation of the Gad system via Mnm pathway–dependent tRNA modification in *E. coli***.** Anaerobiosis and SCFAs activate the Mnm pathway, resulting in mnm^5^s^2^U_34_-modified tRNAs, which fine-tune translation of the transcription factor GadW and thus induce the Gad system. The wobble base U_34_ is modified by several enzymes, producing the predominant mnm^5^s^2^U modifications in specific tRNAs to improve translation efficiency and decoding accuracy of NNA/G codons. Created in BioRender. Four, U. (2026) https://BioRender.com/5h02f8f

**Fig 7:**
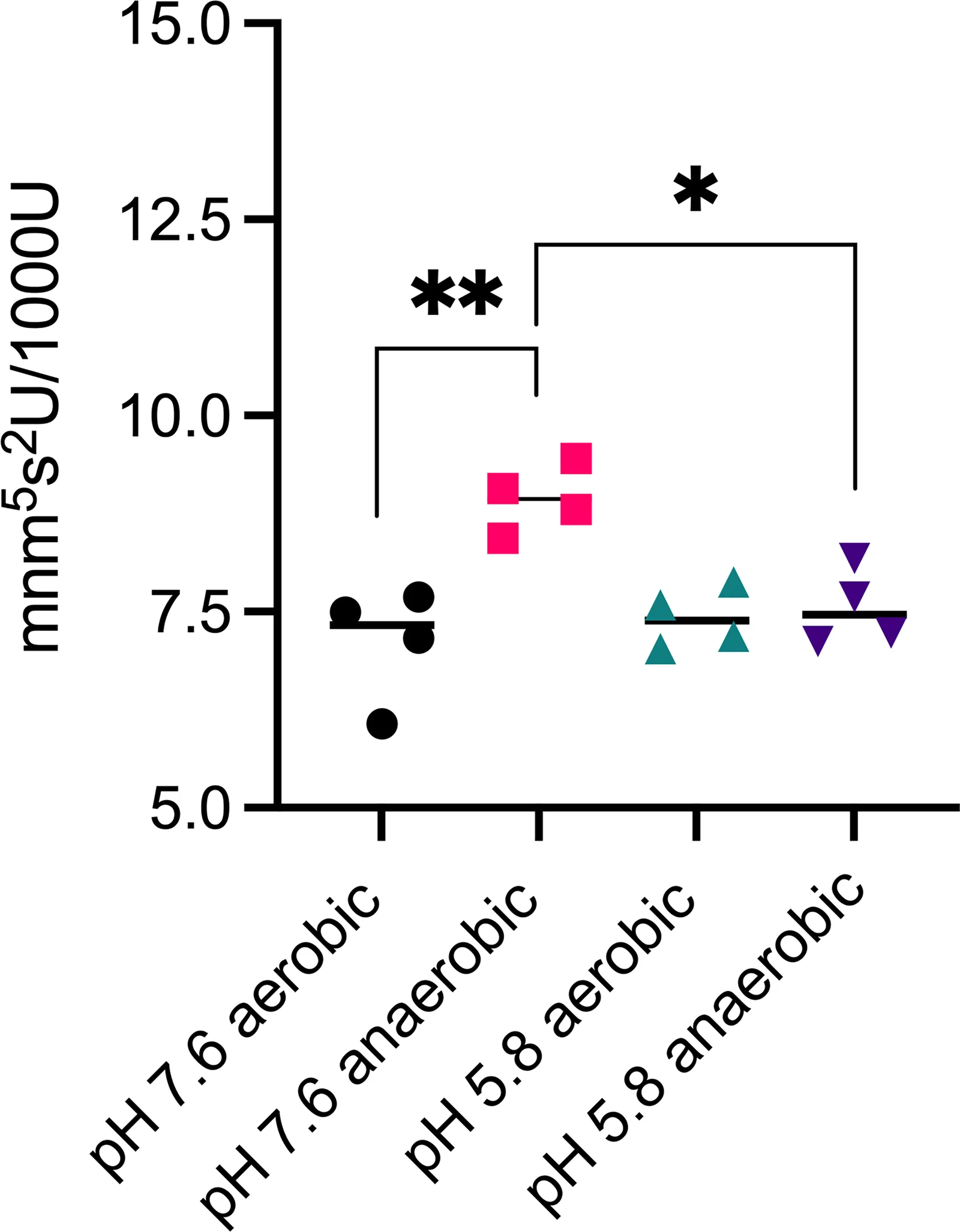
Levels of mnm^5^s^2^U. *E. coli* MG1655 was grown as described in Fig. 2, and cells were harvested after 6 h of growth. tRNA-enriched RNA samples (≤200 nucleotides) were used to assess the mnm^5^s^2^U level by mass spectrometry. The data represent the average of four independent biological replicates. *P*-values were calculated using one-way analysis of variance (ANOVA) and the Tukey multiple comparisons test. Differences were considered statistically significant at thresholds of *P ≤ 0.05 and **P ≤ 0.01.

MnmA and MnmE/MnmG are involved in adding a thiol group (s^2^U_34_ at the 2′ position) and an aminomethyl (nm) or carboxymethylamino (cmnm) group (at the 5′ position) to uridine 34, the wobble position, of tRNAs that recognize distinct codons for Lys, Glu, Gln, Arg, and Gly, all of which read NNA/G codons (36, 48) (see also Fig. 6). The absence of these enzymes has been associated with growth defects under acidic conditions in various bacteria (49–52). Increased levels of pre-tRNA (tRNA precursor) Mnm modifications are associated with survival under acidic stress conditions (52). This prompted us to determine the level of mnm^5^s^2^U in bulk tRNAs prepared from *E. coli* MG1655 under our four test conditions (see Fig. 2) using quantitative mass spectrometry. We detected mnm^5^s^2^U under all four Gad-inducing conditions with a further increase under anaerobic conditions (Fig. 7), in a quantity that exceeded the value described for *Pseudomonas aeruginosa* by a factor of 25 (53). We could not quantify other modifications, such as cmnm^5^s^2^U, cmnm^5^Um, mnm^5^U, and s^2^U, in bulk tRNA because they were below the lower limit of quantification. It should be noted that GadW contains two clusters of three consecutive codons requiring mnm^5^s^2^-modified tRNAs (codons encoding Lys_123_-Lys_124_-Glu_125_, and Lys_170_-Lys_171_-Lys_172_). Such an accumulation of codons requiring mnm^5^s^2^-modified tRNAs is not found in GadE. The data shown in Fig. 6 also indicate the dynamics of the modification state of tRNAs depending on growth and stress conditions. MnmA is an iron-sulfur cluster-containing enzyme (54) whose activity might be sensitive to aerobic and acidic conditions. Nevertheless, further experimentation is required to determine which modified tRNAs/codons are necessary for the fine-tuned translation of acid stress-relieving proteins.

## DISCUSSION

The Gad system provides robust protection against acid stress for both commensal and pathogenic *E. coli* strains, as well as many other bacteria (6, 10, 14, 55, 56). The regulatory network of the Gad system is highly complex, and its activation is influenced by environmental pH, growth phase, and oxygen availability (14, 16, 20). In this study, we systematically analyzed the dynamics of Gad expression in *E. coli* MG1655 during growth in a controlled bioreactor. What began as a control experiment—namely, the cultivation of *E. coli* in buffered minimal medium at pH 7.6—became an ideal setup to study the induction of the Gad system. As cell density increased, dissolved oxygen became limiting, causing *E. coli* to switch to mixed acid fermentation, which lowered the extracellular pH and shifted the culture into the stationary phase (Fig. 1). At the time when the dissolved oxygen level decreased, *gadB*/*gadA* expression began, followed shortly by GadA/GadB synthesis. Moreover, at the same time as oxygen availability decreased, acetic acid and, a little later, formic acid were excreted. In a subsequent experiment, we found that *E. coli* excretes high amounts of acetic acid, resulting in millimolar extracellular concentrations under all Gad-inducing conditions (Fig. 2). A systematic analysis of various organic acids as stimuli for the activation of the Gad system indicated a superior effect not only by acetic acid but also by propionic acid and butyric acid in comparison to HCl or other organic acids, such as succinic acid, formic acid, lactic acid, or citric acid (Fig. 3). Notably, this experiment was conducted at equal extracellular pH values and under conditions that prevented the excretion of acetate. This finding is consistent with previous observations that *E. coli* O157:H7 or *E. coli* K-12 had increased acid tolerance after exposure to SCFAs (46). A further screen for putative regulators involved in SCFAs-sensing identified MnmG/MnmE (Fig. 4). The subsequent proteome profile of MG1655 mutants lacking *mnmA* or *mnmE* revealed a decrease in the level of GadA/GadB and GadW, whereas the level of GadE remained unaffected (Fig. 5). Finally, the highest level of mnm^5^s^2^U_34_ labeled tRNA was found under anaerobic conditions (Fig. 6), coinciding with the increased level of GadW (Fig. 2).

MnmG (also known as GidA) proteins are conserved in Bacteria and Eukarya. GidA (glucose-inhibited division) was first described in *E. coli*, where its disruption affected cell division, but only when cells were grown on glucose (57). It is now well established that MnmG, together with MnmE, catalyzes the addition of the nm or cmnm group at position 5′ of U34 of specific tRNAs (36). Furthermore, there is already evidence that the Mnm pathway is involved in acid resistance (52). Initially, it was described as a pH-independent, glucose-induced pathway that regulates *gadA* and *gadBC* expression by activating *gadE* and controlling *gadA* translation, with the GTPase activity of MnmG being essential for both transcriptional and translational regulation (41). Subsequent studies demonstrated that MnmE is critical for the expression of the *gadE-mdtEF* operon under anaerobic, stationary-phase conditions and can function independently of known regulators such as EvgA, YdeO, RpoS, and GadX, while also showing activity in non-glucose media, contradicting earlier findings (58). Notably, the *mnmE* gene was identified in an unbiased screen as an important gene for the growth of *E. coli* MG1655 under moderate acid stress (51).

We propose the following model. The posttranscriptional modification of uridine at the wobble position (U34) of tRNAs is crucial for optimizing translation (59). A switch of *E. coli* to anaerobiosis may increase the s^2^U thiolation activity of the iron-sulfur cluster-containing enzyme MnmA, which is required for the subsequent nm or cmnm modification of specific tRNAs catalyzed by MnmE/MnmG. This, in turn, may boost the translation of the transcription factor GadW, which activates expression of *gadE*, encoding the master regulator of the Gad system. SCFAs are weak acids that, at low external pH, freely diffuse across the cytoplasmic membrane in their protonated form, leading to their accumulation in the cytoplasm. Although not directly, these organic acids might affect the post-translational modification of proteins. Notably, MnmA was found to be succinylated (60). This hypothetical model is plausible, but requires further investigation.

In summary, the results described here reveal a previously unknown link between SCFAs, tRNA modification, and acid resistance.

## MATERIALS AND METHODS

### Bacterial strains, media, and cultivation conditions

Strain *E. coli* MG1655 was used throughout this study. Strain *E. coli* BW25113 and the mutants from the Keio collections were used for a screen (61). Lysogeny broth (LB) and LB agar were used for strain maintenance and cloning procedures. The Kim-Epstein (KE) medium (100 mM potassium phosphate buffer, 2 mM sodium citrate, 8 mM (NH_4_)_2_SO_4_, 6 µM FeSO_4_, 0.4 mM MgSO_4_ at pH 7.6 or pH 5.8) was used as the standard minimal medium (62). By default, KE medium was supplemented with 0.4% (wt/vol) glucose and 1.5 mM L-glutamic acid; where indicated, glucose was replaced with glycerol (30 mM). Antibiotics were added (if necessary) with the following concentrations: carbenicillin (100 µg/mL) and chloramphenicol (34 µg/mL). Isopropyl β-D-1-thiogalactopyranoside (IPTG) (0.1 mM) was used for the induction of genes of the ASKA plasmids.

For the systematic analysis of the activation profile of the Gad system, a bioreactor (Infors HT Minifors 2, 3 Liter vessel, Rushton impellers, six blades, equipped with inline sensors for pH and pO_2_) was used, which was connected to atmospheric air (air pressure 1.0 bar, rotator 1.5 NI/min), and run at 37°C with 200 rpm. *E. coli* cells were precultured in 500 mL baffled flasks filled with 200 mL glucose KE medium with a starting OD_600_ of 0.2 at 200 rpm and 37°C. After 2-h incubation, cells were harvested by centrifugation (5,000 rpm, 10 min) at 37°C. Then, cells were inoculated into a bioreactor filled with 900 mL KE minimal medium with a starting OD_600_ of 0.05, which was considered as time 0. Samples were collected by centrifugation. The pellets were used for *gadA/gadB* mRNA and GadA/GadB protein analysis, while the supernatants were used for metabolites analysis.

For proteome analysis, extracellular metabolome analysis, and determination of mnm^5^s^2^U tRNA levels, cells were cultivated as follows. *E. coli* MG1655 was cultivated in KE medium supplemented with 0.4% (wt/vol) glucose and 1.5 mM glutamate at either pH 7.6 or pH 5.8. For aerobic conditions, cells were grown in KE medium in 500 mL baffled flasks containing 100 mL at 200 rpm and 37°C. For anaerobic conditions, replicates were grown in 50 mL closed-top-filled Falcon tubes at 37°C. For growth analyses, sample preparation for proteomic and metabolomic analyses, cells were measured every hour and harvested at given timepoints by centrifugation.

Strains, plasmids, and primers used in this study are summarized in Tables 1 to 3.

**TABLE 1:** *E. coli* strains used in this study

Strain	Genotype	Reference
MG1655	F^–^ λ^–^ *ilvG*– *rfb*-50 *rph*-1	(63)
MG1655Δ*mnmA*	In-frame deletion of *mnmA* in MG1655	(52)
MG1655Δ*mnmE*	In-frame deletion of *mnmE* in MG1655	(52)
BW25113	F^-^ λ^-^ Δ(*araD-araB*)567 Δ(*rhaD-rhaB*)568 Δ*lacZ*4787 (::*rrnB*-3) *hsdR*514 *rph*-1	(61)
BW25113Δ*mnmE*	BW25113 *mnmE*::*kan* (JW3684)	(61)
BW25113Δ*mnmG*	BW25113 *mnmG*::*kan* (JW3719)	(61)
BW25113Δ*phoQ*	BW25113 *phoQ*::*kan* (JW1115)	(61)
BW25113Δ*phoP*	BW25113 *phoP*::*kan* (JW1116)	(61)
BW25113Δ*evgA*	BW25113 *evgA*::*kan* (JW2366)	(61)
BW25113Δ*evgS*	BW25113 *evgS*::*kan* (JW2367)	(61)
BW25113Δ*ydeO*	BW25113 *ydeO*::*kan* (JW1494)	(61)
BW25113Δ*gadW*	BW25113 *gadW*::*kan* (JW3483)	(61)
BW25113Δ*gadX*	BW25113 *gadX*::*kan* (JW3484)	(61)
BW25113Δ*gadE*	BW25113 *gadE*::*kan* (JW3480)	(61)

**TABLE 2:** Plasmids generated and used in this study

Plasmid	Genotype	Reference
pCA24N	pMB1 replication origin, high copy number, lacI^q^, Cm^R^	(64)
pCA24N-*gadW*	pCA24N p_T5-lac_:*gadW*	(64)
pCA24N-*gadE*	pCA24N p_T5-lac_:*gadE*	(64)
pCA24N-*mnmG*	pCA24N p_T5-lac_:*mnmG*	(64)
pBBR1-MCS4	Broad-host-range cloning vector, *mob* region for conjugative transfer, Amp^R^	(65)
pBBR1-MCS4- P*gadA-egfp*-P*gadB-mCherry*	Promoter region of *gadB* (−288 to −1 bp) controlling expression of mCherry (BamHI and SacI) and the promoter region of *gadA* (−180 to −1 bp) controlling expression of *egfp* (BamHI and EcoRI) in pBBR1-MCS4*,* arranged in opposite orientations, Amp^R^	This study

**TABLE 3:** Primers used in this study^*a*^

Primer name	Sequence 5′−3′
*gadA*-180-F-BamHI	tagcc**ggatcc**ggcgatttttattacgataa
*gadA*-Pro-egfp-OL-F	ataaatttaaggagttcgaaatgcgtaaaggagaagaact
*gadA*-Pro-egfp-OL-R	agttcttctcctttacgcatttcgaactccttaaatttat
*gadB*-288-F-BamHI	tagcc**ggatcc**tgcgttcaaaataataatca
*gadB*-Pro-mCherry-OL-F	atcattttaaggagtttaaaatggtgagcaagggcgagga
*gadB*-Pro-mCherry-OL-R	tcctcgcccttgctcaccattttaaactccttaaaatgat
*gadA*-pro-mCherry-ol-f	ataaatttaaggagttcgaaatggtgagcaagggcgagga
*gadA*-pro-mCherry-ol-r	tcctcgcccttgctcaccatttcgaactccttaaatttat
*egfp*-rev-EcoRI	tagcc**gaattc**ttatttgtatagttcatcca
*mCherry*-rev-SacI	tagcc**gagctc**agcttttacttgtacagctcgtcc
*gadA_*qPCR fwd	ggcgcaaaggccatttctactatcg
*gadA_*qPCR rev	cgagcgttgccatcaagatataattc
16S_for_qPCR	cgaacggtaacaggaagaag
16S_rev_qPCR	gcacatccgatggcaagagg

^*a*^ Sites for restriction enzymes are indicated in bold.

### Generation of plasmids

All the enzymes and biology kits were used by following the standard protocols from the manufacturers. Genomic DNA of *E. coli* MG1655 was extracted using the Hi Yield gDNA Mini Kit (SLG, Gauting). Plasmid extraction was performed by using the Hi Yield Plasmid Mini Kit (SLG, Gauting). DNA fragments of the promoters of the target genes (*gadA* and *gadB*) were amplified by PCR with the specific primers (Table 3) and the template genomic DNA of *E. coli* MG1655, and purified from agarose gels using the Hi Yield PCR Cleanup and Gel Extraction Kit (SLG, Gauting). The DNA fragments of *egfp* and *mCherry* were amplified from a triple reporter cassette (66). All fragments of gene fusion were generated by using overlap PCR, followed by digestion with the respective restriction enzymes and ligated with T4 DNA ligase into the pBBR1-MCS4 plasmid (65).

### qRT-PCR

RNA was purified using the phenol-chloroform-isoamyl alcohol (PCI) protocol (67) with modifications. RNA concentration and purity were checked using a Nanodrop spectrophotometer (ND-1000, PeqLab, Germany). The RNA samples were then treated with the TURBO DNA-free Kit (Invitrogen, Germany) to remove genomic DNA. The quality and concentration of RNA samples were measured again with the Nanodrop spectrophotometer. Complementary DNA (cDNA) was synthesized from 2 µg of total RNA using the iScript Advanced Script (Bio-Rad) according to the manufacturer’s protocol. Then, 3 µL of the 1:10 diluted cDNA mixed with 0.75 µL forward primer, 0.75 µL reverse primer, 7.5 µL of the 2x-SsoAdvanced Univ SYBR Green Supermix (Bio-Rad), and 3 µL of the RNase-free water were dispensed into a 96-well PCR plate (Bio-Rad). The qPCR was performed in a Bio-Rad CFX real-time cycler. The ribosome gene of 16S rRNA was used as the internal reference to analyze the qRT-PCR data according to the ΔΔCt method (68). To keep the Ct value in the same range, the template cDNA used to measure the 16S rRNA gene was diluted 10,000-fold compared to the sample used to measure *gadA/gadB* mRNA. The primer efficiency was calculated using a serial dilution; the results were as follows: 16S rRNA (91.47%) and *gadA/gadB* mRNA (90.01%). The melting curves were evaluated to assess the specificity of the amplification. The primer sequences used in this study are listed in Table 3.

### Quantitative western blotting

The collected cells were adjusted to an OD_600_ of 10. Then, 16 µL of cells was mixed with 4 µL 5 x loading buffer and boiled for 5 min at 100°C. Proteins were separated by 12.5% (wt/vol) sodium dodecyl sulfate (SDS) polyacrylamide gel electrophoresis (PAGE) (69). Proteins from the SDS-polyacrylamide gels were transferred to nitrocellulose membranes (Amersham, GE Healthcare) using wet blotting. The specific anti-GadA/GadB antibody (Eurogentec) was used to detect GadA/GadB using a 1:5,000 dilution in 3% (wt/vol) bovine serum albumin solution in 1× TBS buffer (10 mM Tris/HCl pH 7.5, 500 mM NaCl, 0.05% [vol/vol] Tween 20, 0.2% [vol/vol] Triton X-100). The 1× TBS buffer and 1× TBST buffer (1× TBS buffer, 0.05% [vol/vol] Tween 20) were used for washing steps. The Donkey Anti-rabbit IgG H&L (IRDye 680RD) preadsorbed (Abcam, ab216779) was used as the secondary antibody at a 1:20,000 dilution. The Odyssey CLx imaging system (LI-COR Biosciences) was used for imaging and data analysis. The relative amount of GadA/GadB was normalized to the reference protein, purified His-tagged GadA.

To assess relative GadW abundance in *E. coli* MG1655 wild type and the *mnmA* and *mnmE* mutant strains, cells were transformed with the expression plasmid pCA24N-*gadW*, encoding His-tagged GadW. Cultures were anaerobically grown in KE medium at pH 5.8 supplemented with 0.4% (wt/vol) glucose and 1.5 mM L-glutamic acid. After 3 h of growth, expression was induced by the addition of 100 µM IPTG, and cells were harvested 1 h post-induction. Protein samples were separated by SDS-PAGE and transferred to a membrane for immunodetection. Membranes were blocked for 1 h in TBS containing 0.3% (wt/vol) BSA and subsequently incubated for 1 h with mouse anti-His tag antibody (Bio-Rad, MCA139D550, DyLight550) and anti-GAPDH antibody (Invitrogen, MA5-15738-D680, DyLight680), each at a 1:5,000 dilution. All incubation steps were performed protected from light. Membranes were washed twice with TBS containing 0.05% (vol/vol) Tween-20 and twice with TBS, and fluorescence signals were recorded using the ChemiDoc Imaging System (Bio-Rad). Band intensities were quantified in ImageJ (70) and normalized to GAPDH as a loading control.

### High-performance liquid chromatography

Quantification of the organic compounds, such as acetate, formate, lactate, and succinate, was performed by high-performance liquid chromatography (HPLC, Prominence-i LC-2030C, Shimadzu, Kyoto, Japan) equipped with an ion exchange column (Aminex HPX-87H 300 mm × 7.8 mm, Bio-Rad, Hercules, CA, USA) and a refractive index detector (RID-20A, Shimadzu, Kyoto, Japan). For the analysis, an isocratic flow rate of 0.6 mL min^−1^ with 5 mM H_2_SO_4_ as the mobile phase and a temperature of 60°C was applied. 10 µL of the sample was injected. External standards were used to determine the concentration of organic compounds.

### Quantification of short-chain fatty acids and organic acids by mass spectrometry

Cells were grown under aerobic and anaerobic conditions at pH 5.8 and pH 7.6, respectively (see bacterial strains, media, and cultivation conditions). Supernatants were harvested at time points 4, 6, 8, and 10 h, and cells were removed by centrifugation for 10 min at 3,000 × *g*. Supernatants were sterile filtered using a sterile PVDF syringe filter (0.22 µM pore size, Carl Roth). 20 µL of culture supernatant was extracted with 80 µL acetonitrile. After 2 min of shaking at 10°C and 2,000 rpm using an Eppendorf ThermoMixer (Eppendorf, Hamburg, Germany), the samples were centrifuged for 10 min at 10°C at 15,000 rpm. 40 µL of the clear supernatant was used for the 3-nitrophenylhydrazin (3-NPH) derivatization (71). Briefly, 10 µL of ca. 50 µM internal standard solution, 20 µL 120 mM EDC HCl-6% pyridine solution, and 20 µL of 200 mM 3-NPH solution were added. After shaking for 30 min at 40°C, 910 µL of acetonitrile/water (50:50, vol:vol) was added. After centrifugation for 2 min at 10,000 rpm, the supernatant was transferred to a LC-MS/MS vial for mass spectrometric analysis. The measurement was performed using a QTRAP 5500 triple quadrupole mass spectrometer (Sciex, Darmstadt, Germany) coupled to an ExionLC AD (Sciex, Darmstadt, Germany) ultrahigh performance liquid chromatography system. The electrospray voltage was −4,500 V, curtain gas 35 psi, ion source gas 1 55 psi, ion source gas 2 65 psi, and the temperature 500°C. The MRM parameters were optimized using commercially available standards (72). The chromatographic separation was performed on a 1.7 μm, 100 × 2.1 mm, 100 Å Kinetex C18 column (Phenomenex, Aschaffenburg, Germany) with 0.1% formic acid (eluent A) and 0.1% formic acid in acetonitrile (eluent B) as elution solvents. An injection volume of 1 µL and a flow rate of 0.4 mL/min were used. The gradient elution started at 23% B, which was held for 3 min; afterward, the concentration was increased to 30% B at 4 min, with another increase to 40%B at 6.5 min. At 7 min, 100% B was used, which was held for 1 min; at 8.5 min, the column was equilibrated at starting conditions. The column oven was set to 40 °C. Data acquisition and instrumental control were performed with Analyst 1.7 software (Sciex, Darmstadt, Germany). The data were analyzed with MultiQuant 3.0.3 (Sciex, Darmstadt, Germany).

The mass spectrometry metabolomics data have been deposited under the MassIVE accession number MSV000101258.

### Quantification of the mnm^5^s^2^U34-tRNA level

Cells were harvested, and RNA was isolated using the PCI protocol as described before (73). This was followed by treatment with RNase-free DNase I (New England Biolabs) according to the manufacturer’s instructions. From total RNA, ≤200 nt RNA samples were separated using a size-selection protocol according to the manufacturer’s instructions RNA Clean & Concentrator Kit (Zymo Research). The quality, integrity, and size of the RNA were assessed via chip gel electrophoresis using a 2100 Bioanalyzer and an RNA Nano Chip Kit (Agilent Technologies). Quantitative mass spectrometry analysis was performed as previously described (52, 73). The nucleoside 5-methylaminomethyl-2-thiouridine (mnm^5^s^2^U) was provided by the Susanne Häußler laboratory for calibration. The absolute abundance of modified nucleosides was calculated as the abundance per 1,000 of the canonical uracil.

### Reporter activity assay

The activity of promoter fusions in *E. coli* was measured using a TECAN Spark 20 M plate reader or an Infinite 200Pro plate reader by recording the optical density (OD_600_) and fluorescence intensity in a 96-well plate at 37°C with orbital shaking for 3 h. The data were expressed as relative fluorescence units (RFU), defined as fluorescence units divided by optical density at OD_600_. The eGFP fluorescence intensity was measured with an excitation wavelength of 485 nm and an emission wavelength of 535 nm. The mCherry fluorescence intensity was measured with an excitation wavelength of 560 nm and an emission wavelength of 612 nm.

### Proteomics

Cells were grown under aerobic and anaerobic conditions at pH 5.8 and pH 7.6, respectively (see bacterial strains, media, and cultivation conditions). Harvested cell pellets were disrupted using cell disruption buffer (6 M guanidine-HCl, 50 mM Tris-HCl, 0.5 mM PMSF, 10 mM DTT) by incubation at 40°C for 30 min. The resulting supernatants were collected by centrifugation for 10 min at 3,000 × *g*, and protein concentrations were determined using Pierce 660 reagent (Thermo Scientific) according to the manufacturer’s instructions. Albumin standard (Thermo Scientific) in ultrapure water was used for calibration. A total protein amount of 100 µg was used for further proteomic analysis.

Protein samples were processed using the Single-Pot, Solid-Phase-enhanced Sample Preparation (SP3) protocol with minor modifications (74). Carboxylate-modified paramagnetic beads (Cytiva SpeedBeads) were used at a 1:1 ratio. Protein samples (100 µg) were reduced with 10 mM dithiothreitol (DTT) for 30 min at 37°C and alkylated with 15 mM iodoacetamide for 30 min at room temperature in the dark. Alkylation was quenched with 5 mM DTT for 10 min. A 10:1 bead-to-protein ratio (wt/wt) was applied, and ethanol was added to a final concentration of 50% (vol/vol). Samples were incubated for 10 min at room temperature for protein binding, and beads were washed four times with 80% (vol/vol) ethanol. After the final wash, beads were air-dried for 1 min under a fume hood. Beads were resuspended in 100 µL 100 mM ammonium bicarbonate (ABC), and trypsin digestion was performed overnight at 37°C (enzyme-to-substrate ratio 1:25). Following digestion, the supernatant was collected, beads were rinsed with 60 µL 100 mM ABC, and both supernatants were combined. The digest was acidified to pH 3 with formic acid and centrifuged at 14,000 × *g* for 10 min. Peptides were desalted using C18 StageTips (75).

Liquid chromatography−tandem mass spectrometry (LC-MS/MS) was performed on a nano-LC system (nanoElute2, Bruker Daltonics) coupled to a timsTOF HT mass spectrometer (Bruker Daltonics) using a CaptiveSpray nano electrospray ionization (ESI) source. The nano-LC system was equipped with a PepSep Ultra separation column (C18, 25 cm × 75 µm × 1.5 µm, Bruker Daltonics). The peptide mixture (500 ng) was separated over a 60-min linear gradient of 5%–37% (vol/vol) acetonitrile at a constant flow rate of 250 nL/min. The column was kept at 50°C in a column oven throughout the run. Data-independent acquisition (DIA) was performed using the following settings: mass range: 100–1,700 m/z, ion mobility range: 0.85–1.3 V*s/cm^2^, ramp time: 100 ms.

Raw files were processed using DIA-NN v2.0 (76). A library-free search was performed against the *E. coli* reference proteome (UP000000625_2025_04_24, Uniprot, www.uniprot.org) using DIA-NN default settings unless otherwise specified. Trypsin was specified as the proteolytic enzyme, allowing a maximum of one missed cleavage, and common contaminants were included in the search. Carbamidomethylation of cysteines was set as a fixed modification. Match-between-runs was disabled. Precursor and fragment mass accuracies were set to automatic inference. False discovery rate (FDR) was controlled at 1% at both the precursor and protein-group levels using the DIA-NN target-decoy approach. Protein quantification was based on DIA-NN MaxLFQ intensities. GadA and GadB proteins were manually grouped due to their high sequence identity. Protein abundances correspond to log_2_-transformed DIA-NN MaxLFQ intensities.

Downstream statistical analysis was performed using Perseus version 2.0.11 (77), and graphs were prepared with R and RStudio v4.4.1 (78, 79). All biological replicates were grouped, and log_2_ LFQ intensities were filtered to retain protein groups with non-missing quantified LFQ values in at least two of the three replicates in at least one experimental group. Protein-group identifications were controlled at 1% FDR in DIA-NN. To enable statistical evaluation, missing values were imputed with random numbers drawn from a normal distribution.

The mass spectrometry proteomics data have been deposited to the ProteomeXchange Consortium via the PRIDE partner repository with the data set identifier PXD076108.

## Data Availability

The mass spectrometry metabolomics data have been deposited under the MassIVE accession number MSV000101258.

## SUPPLEMENTAL MATERIAL

Data set S1 jb.00285-26-s0001.xlsx Proteome data.

ASM does not own the copyrights to Supplemental Material that may be linked to, or accessed through, an article. The authors have granted ASM a non-exclusive, world-wide license to publish the Supplemental Material files. Please contact the corresponding author directly for reuse.
